# Supplementary material for: Lim Domain Binding 3 (Ldb3) Identified as a Potential Marker of Cardiac Extracellular Vesicles
Source: Int J Mol Sci. 2022 Jul 1;23(13):7374. doi: 10.3390/ijms23137374 (PMC9266879; doi:10.3390/ijms23137374)
Supplement: Supplementary file 1 [file ijms-23-07374-s001.zip › ijms-1764779-supplementary.pdf]

International Journal of
Molecular Sciences

MDPI

Article

LIM Domain Binding 3 (Ldb3) identified as a potential marker of cardiac extracellular vesicles

Fadi Abou Zeid[#1], Henri Charrier[#1], Olivia Beseme[1], Jean-Baptiste Michel[2], Paul Mulder[3], Philippe Amouyel[1], Florence Pinet[1*] and Annie Turkieh[1*]

[1] Univ. Lille, Inserm, CHU Lille, Institut Pasteur de Lille, U1167 - RID-AGE - Facteurs de risque et déterminants moléculaires des maladies liées au vieillissement, F-59000 Lille, France; fadi.abou-zeid@pasteur-lille.fr (F.A.); henri.charrier@genoscreen.fr (H.C.); olivia.beseme@pasteur-lille.fr (O.B.); philippe.amouyel@pasteur-lille.fr (P.A.); florence.pinet@pasteur-lille.fr (F.P.); ani.turkieh@pasteur-lille.fr (A.T.)

[2] Université de Lorraine, Inserm, U1116 - DCAC, 54000 Nancy, France; jean-baptiste.michel@inserm.fr

[3] Normandie Univ, UNIROUEN, Inserm U1096, FHU-REMOD-HF, 76000 Rouen, France; paul.mulder@univ-rouen.fr (P.M.); vincent.richard@univ-rouen.fr (V.R.)

[#] Co-first-authors

* Correspondence: A.T: ani.turkieh@pasteur-lille.fr; +33 (0)3 20 87 73 62; FP: florence.pinet@pasteur-lille.fr; +33 (0)3 20 87 72 15

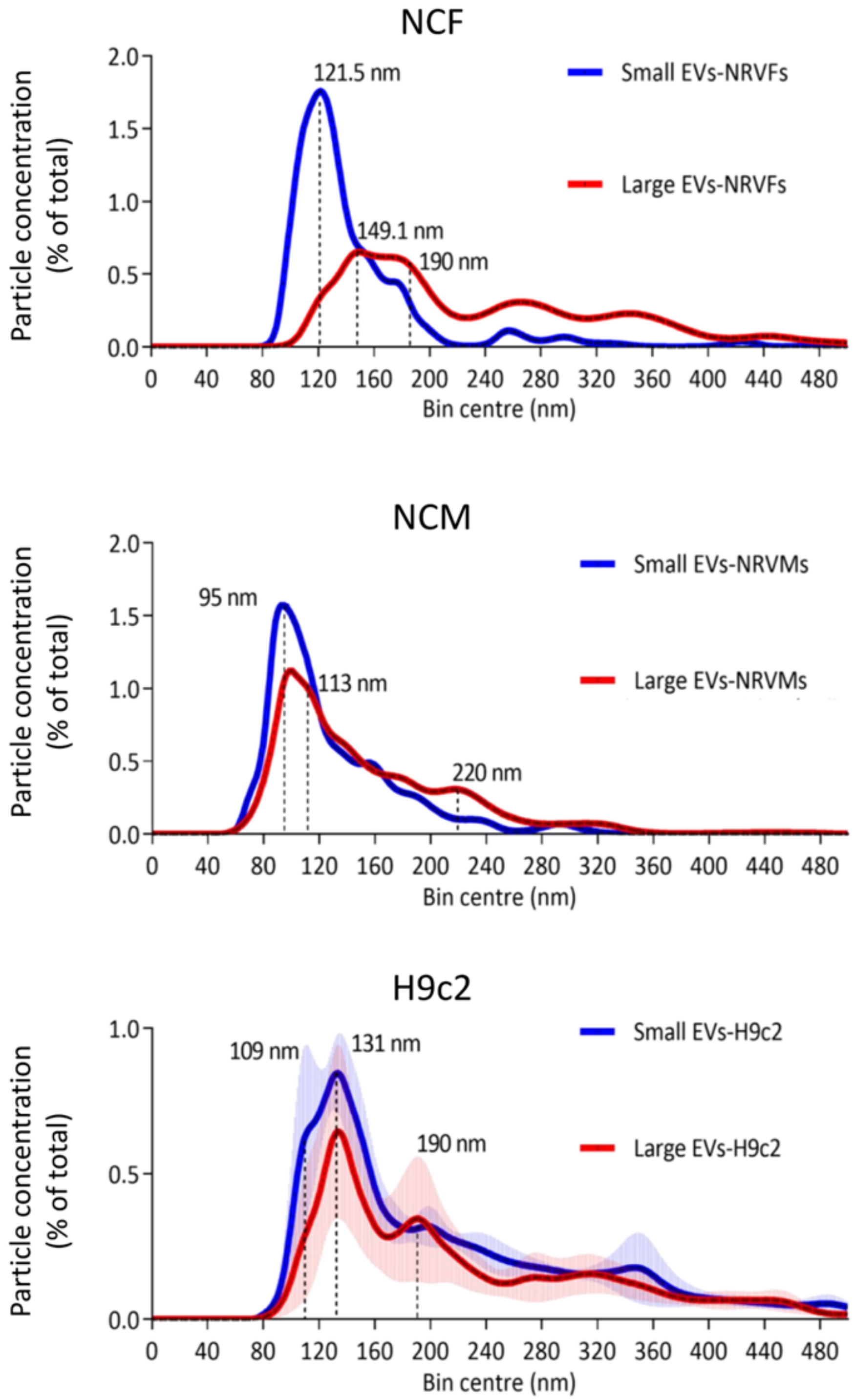

Figure S1. Particle size distribution of EVs by nanoparticle tracking analysis (NTA). They were measured by NTA (Malvern, UK) for EVs isolated from NCF (top), NCM (middle), and H9c2 (bottom) conditioned culture media. NCF and NCM measurements correspond to one sample. H9c2 measurement corresponds to the mean ± SE (shadings) of 3 different preparations.

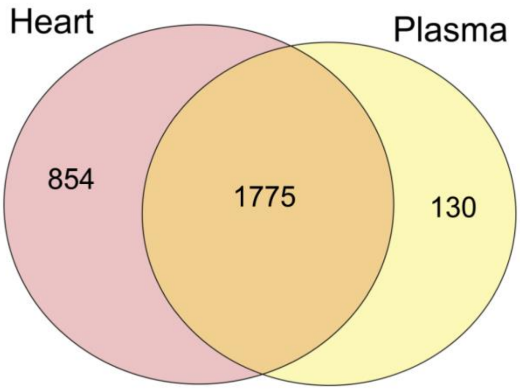

Figure S2. Venn diagram showing the number of cardiac- and plasma-EV proteins identified by LC-MS. Only proteins detected in at least 3 out of 4 samples in each group were used.

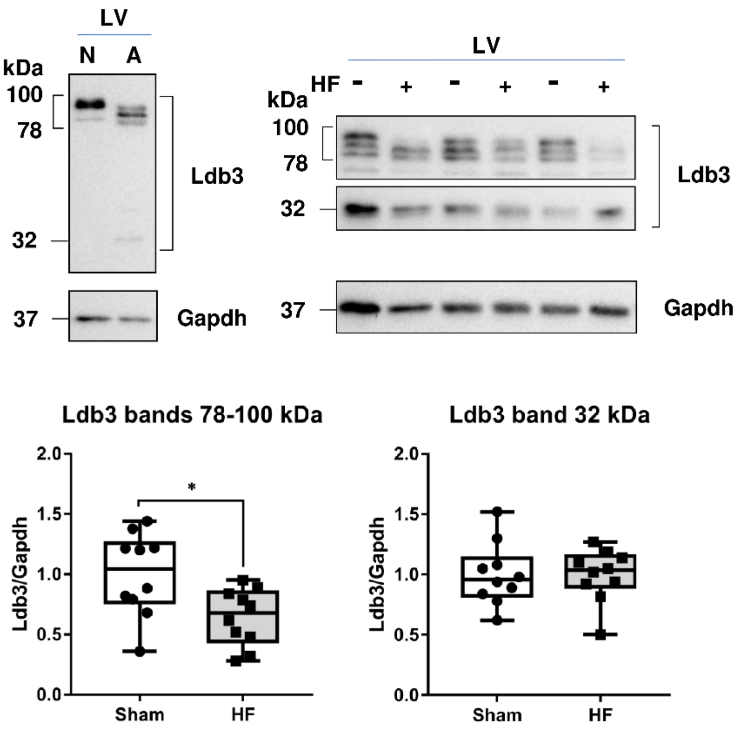

Figure S3. Detection of Ldb3 isoforms. Different isoforms were detected in neonate (N) and adult (A) ratLV tissue (top left panel), and in adult LV tissue from adult sham- (-) and HF- (+) rats (top right panel and bottom panels). Data are expressed as median with interquartile ranges. *$P< 0.05$.
